# Crystal Structures and Electronic Properties of BaAu Compound under High Pressure

**DOI:** 10.3390/ma15207381

**Published:** 2022-10-21

**Authors:** Bingtan Li, Jianyun Wang, Shuai Sun, Hanyu Liu

**Affiliations:** 1 State Key Laboratory of Superhard Materials, International Center of Computational Method & Software, College of Physics, Jilin University, Changchun 130012, China; 2 Engineering Training Center, Jilin University, Changchun 130012, China

**Keywords:** Au-bearing alloy, high pressure, first-principles calculation

## Abstract

The investigations of Au-bearing alloy materials have been of broad research interest as their relevant features exhibit significant advantages compared with pure Au. Here, we extensively investigate the compression behaviors of BaAu compounds via first-principles calculations and find that a high-pressure cubic phase is calculated to be stable above 12 GPa. Further electronic calculations indicate that despite the low electronegativity of Ba, *Fd*-3*m-*structured BaAu exhibits metallic characteristics, which is different from those of semiconducting alkali metal aurides that possess slight characteristics of an ionic compound. These findings provide a step toward a further understanding of the electronic properties of BaAu compounds and provide key insight for exploring the other Au-bearing alloy materials under extreme conditions.

## 1. Introduction

Gold (Au), one of the most well-known precious metals, has attracted significant interest due to its chemical inertness and fascinating physical properties under normal conditions [1,2,3,4,5,6,7]. Furthermore, it has been found that Au could react with other elements and form plenty of aurides with diverse applications such as catalysis, electronic transport, and optics [8,9,10,11]. Among them, Au-based alloys have become an important family of materials because of their excellent performances, superior to pure Au. For instance, the economic cost and stability are simultaneously balanced in Au-Cu alloys [12,13,14]; superconductivity that is inaccessible in Au has been observed in Au-In and Au-Pb alloys [15,16,17]; Au and Li atoms can form unexpected stoichiometric intermetallic compounds at high pressure, where Au obtains electrons from Li and behaves as a *p*-block element [18]. Therefore, it is of great interest to further investigate new gold-based alloy materials that may possess remarkable properties.

It is worth mentioning that some alkali metal aurides, including KAu, RbAu, and CsAu, are semiconductors [19,20,21,22], whose bandgaps range from 0.1 eV for KAu to 1.0 eV for CsAu crystallizing in a CsCl-type structure [20], albeit the fact that all the constituent elements are typical metals. This anomaly can be understood as alkali metals with relatively low electronegativities dramatically donating valence electrons to Au and showing ionic-compound-like features in the forming compounds. Compared to alkali-metal elements, alkaline earth metals have similar low electronegativity, ionic radii, and -6*s* orbital energy [23,24,25]. Among alkaline earth metals, Ba has the lowest electronegativity based on the Pauling scale, which indicates that it is easy to lose electrons and tends to become a cation once its compound forms [25]. Previous studies [26] indicate that the BaAu compound was stabilized with *Pnma* symmetry at atmospheric pressure, which is also theoretically suggested in our recent study [27]. However, the ground-state *Pnma*-structured BaAu is still metallic material under ambient conditions [28].

Moreover, it has been recognized that pressure is an effective tool to shorten chemical bonds and reconstruct atomic arrangements, thus inducing a crystal structure phase transition and the appearance of novel electronic structures inaccessible at atmospheric pressure [29,30,31,32,33,34,35]. For instance, the typical alkali Na metal has been experimentally proven to become a transparent insulator with a large bandgap under high pressure due to the *p*-*d* orbital hybridization of shell electrons and the electron localization in atomic interstices [36]. The compressed NiTi intermetallic compound is proposed to be a semiconducting state associated with its unique band structure and the nature of 3*d* orbitals localization [37]. As a consequence, it is an open question to explore whether a similar property could appear in any other system other than alkali metal aurides and whether BaAu could be a semiconductor under high-pressure.

In this work, therefore, we systematically conduct a theoretical investigation of stochiometric BaAu and its related electronic properties under high pressure. Our first-principles calculations reveal that the compressed BaAu compound is still metallic below megabar pressure as only part of the valence electrons of Ba are transformed to Au, and the residual ones are still cruising electrons. These findings uncover the high-pressure properties of the BaAu intermetallic compound and provide key insights for exploring the other Au-bearing alloy materials under extreme conditions.

## 2. Computational Details

**Figure 1:**
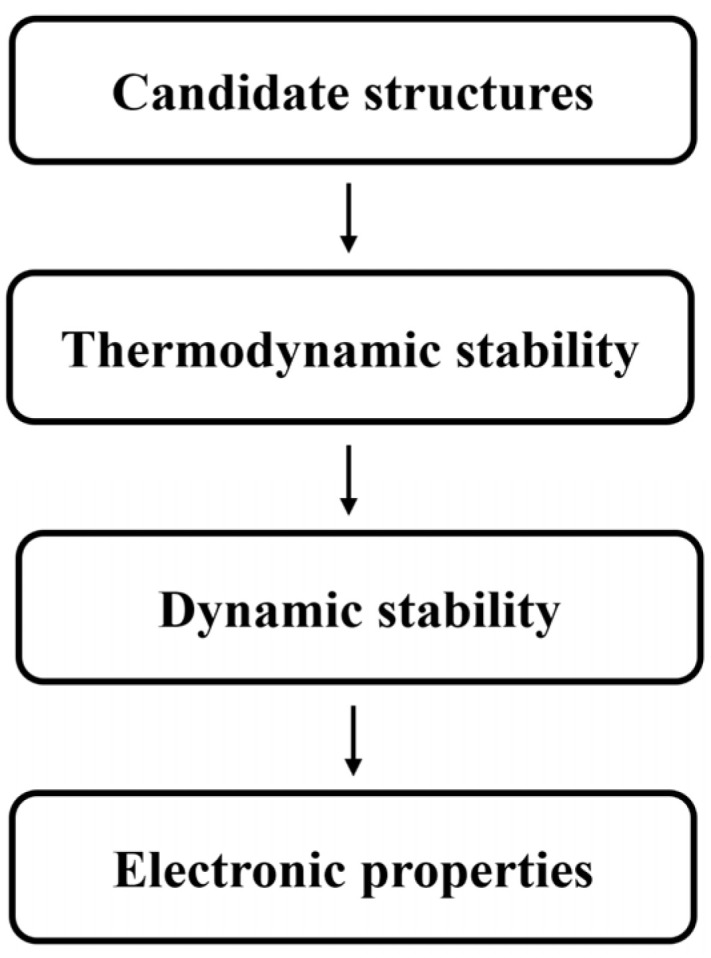
The schematic figure of this manuscript.

First-principles calculations, including total energy calculations, structural relaxations, electronic structures, and phonon dispersions for the BaAu structures under different pressures, were performed using the projector augmented-wave [38] method in the Vienna *Ab initio* Simulation Package (VASP) code Version 6.1.0 [39]. The Perdew–Burke–Ernzerhof (PBE) [40] in the generalized gradient approximation (GGA) was used to describe the exchange-correlational functional [41]. The electron–ion interactions were represented by pseudopotentials built within the scalar relativistic projector augmented wave method with 5*s*^2^5*p*^6^6*s*^2^ and 5*d*^10^6*s*^1^ valence electrons for Ba and Au atoms, respectively. A plane-wave kinetic energy cutoff of 600 eV and dense *k*-point sampling (2*π* × 0.03 Å^−1^) was shown to give an excellent convergence of energies. The electron localization function (ELF) [42,43] was also calculated using VASP code. The dynamical stabilities of the ground-state structures at varying pressures were determined by using the density functional perturbation theory (DFPT) [44] as implemented in the Phonopy code [45,46,47] with a 600 eV energy cutoff. A 2 × 3 × 2 supercell with 96 atoms and a 3 × 3 × 3 supercell with 108 atoms were used to calculate the phonon dispersions for an ambient *Pnma*-BaAu structure and high-pressure *Fd*-3*m*-BaAu under high pressure, respectively. In this process, force was calculated with a threshold of 1 × 10^−7^ eV/Å. The schematic figure of this manuscript was shown in Figure 1.

## 3. Results and Discussion

**Figure 2:**
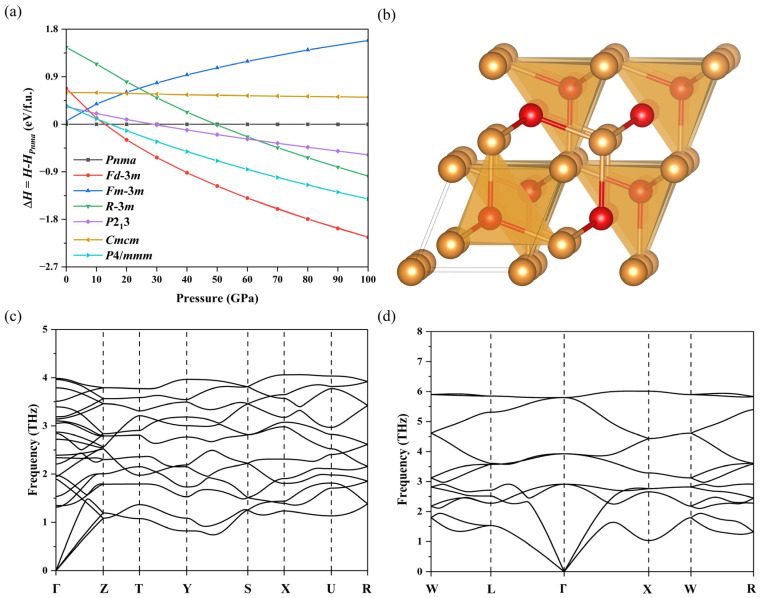
(**a**) Enthalpy as a function of external pressure for selected structures of BaAu in different symmetries. (**b**) The crystal structure of the *Fd*-3*m* BaAu under high pressure. Yellow and red spheres represent Ba and Au atoms, respectively. Phonon dispersions of BaAu with (**c**) *Pnma* symmetry at 10 GPa and (**d**) *Fd*-3*m* symmetry at 25 GPa.

To study the thermodynamic stability of intermetallic compound BaAu at high pressure, AB-type prototypes of binary Au-bearing compounds selected from the Open Quantum Materials Database (OQMD) [48,49] were chosen as candidate structures. The enthalpies of these structures were calculated as a function of the external pressure and plotted relative to the enthalpy of the ambient *Pnma*-BaAu structure, as shown in Figure 2a. Under a pressure range from 0 to 12 GPa, *Pnma*-BaAu was the lowest-energy structure. At elevated pressures above 12 GPa, the *Fd*-3*m* structure became energetically more favorable than *Pnma*-BaAu; therefore, the *Pnma*-BaAu transformed into *Fd*-3*m-*BaAu at high pressure. At atmospheric pressure, the lattice parameter of *a*/*b* and *a*/*c* was ~1.69 and 1.30, respectively. Under high pressure, the *Pnma*-BaAu was drastically compressed, the lattice parameter of *a* and *c* showed a rapid decrease, and a structural phase transition occurred at ~12 GPa, when the *Pnma*-BaAu structure transformed into a cubic structure with *Fd*-3*m* symmetry. This high-pressure *Fd*-3*m* phase consisted of two interpenetrating {BaAu_4_} and {Ba_4_Au} tetrahedra (Figure 2b), whose lattice parameters and atomic coordinates are listed in Table 1. Table S1 gives detailed structural information of other BaAu compounds in the Supplementary Materials. The phonon dispersion spectra of ambient and high-pressure structures were simulated based on density functional perturbation theory (Figure 2c,d), where there is no imaginary phonon mode in the first Brillouin zones. These results confirmed the dynamical stability of these two phases.

**Figure 3:**
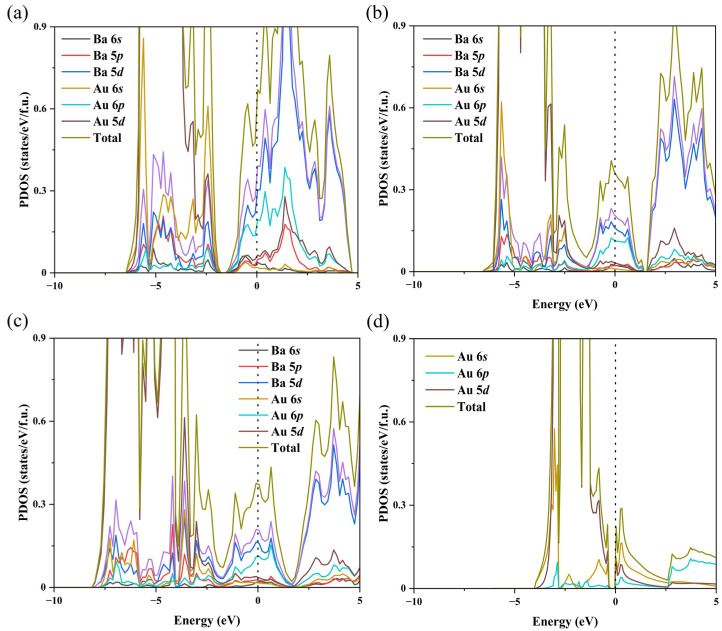
(**a**) PDOS calculated using the Perdew–Burke–Ernzerhof functional for BaAu in the *Pnma* structure at 10 GPa. PDOS calculated for BaAu in the *Fd*-3*m* structure at (**b**) 25 GPa, (**c**) 100 GPa, and (**d**) hypothetical Ba_0_Au at 25 GPa. The dashed line at zero indicates the Fermi energy.

To provide insights into the nature of the chemical bonding of the BaAu compounds, we calculated the projected density of states (PDOS) of the stable phases at 10 (a), 25 (b), and 100 GPa (c). In Figure 3b,c, *Fd*-3*m*-BaAu showed a significant Au 5*d* component below the Fermi level. By analyzing the electronic states around the Fermi level, we found considerable overlap between Ba 5*d* and Au 6*p* orbits, indicating that the charge transfer and the components participated in the Ba-Au bonding. Furthermore, a hypothetical model system of Ba_0_Au was constructed, in which all the Ba atoms were removed from the high-pressure *Fd*-3*m*-BaAu structure. The calculated PDOS of Ba_0_Au is plotted in Figure 3d. The absence of 6*p* electrons in the hypothetical Ba_0_Au structure confirmed that Au 6*p* orbital gained electrons from Ba in BaAu compounds. These results also indicated that the Ba 5*d* orbital had a higher energy relative to Ba 6*s* under current pressure, which has been demonstrated in previous studies [23]. In addition, the calculated PDOS of BaAu in the *Pnma* structure is shown in Figure 3a. The structure of orthorhombic *Pnma*-BaAu showed metallic characteristics at 10 GPa.

**Figure 4:**
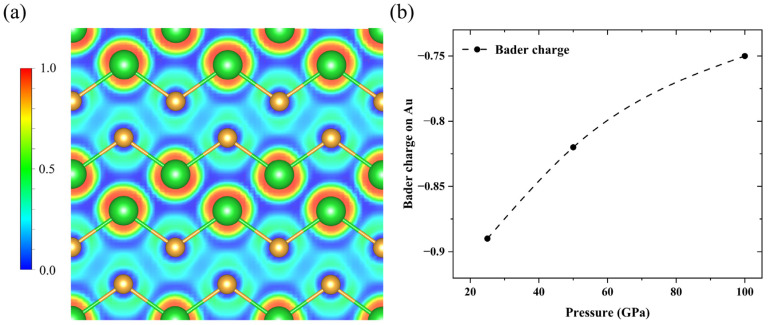
(**a**) Calculated electron localization function (ELF) for *Fd*-3*m*-BaAu compounds on the (101) plane at 25 GPa. (**b**) Pressure dependence of the Bader charges on Au in *Fd*-3*m-*structured BaAu from 25 to 100 GPa.

Moreover, we calculated the electron localization function (ELF) to investigate the chemical bonding in the crystal structure of BaAu under high pressure. As shown in Figure 4a, the low values between Ba and Au atoms showed the ionic bonding feature of Ba-Au bonds, implying the charge transfer between Ba and Au atoms. To further illustrate the charge transfer between Ba and Au atoms, we performed Bader charge analysis on BaAu compounds. As shown in Figure 4b, it is obvious that the Bader charge on Au decreased with increasing pressure. The Bader charge on Au demonstrates a Ba^1+^ oxidation state in high-pressure BaAu compounds. The accepted charges from Ba mainly filled the 6*s* and 6*p* orbitals of Au, which plays an important role in stabilizing BaAu compounds under pressure. Moreover, considering the bond length of Ba-Au bonds in *Fd*-3*m*-BaAu compounds, the interaction between Ba and Au atoms was examined by calculating the integrated crystal orbital Hamilton populations (ICOHP), which can be scaled with the bond strength in compounds by counting the energy-weighted population of wavefunctions between two atomic orbitals. The calculated ICOHP of Ba-Au bonds were −0.18 and −0.17 eV per pair in BaAu compounds at 25 and 100 GPa, respectively, indicating the strengthening of Ba-Au interactions.

**Figure 5:**
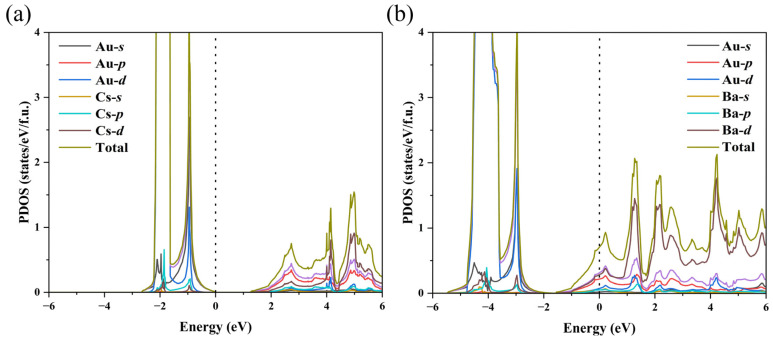
PDOS calculated for CsAu (**a**) and BaAu (**b**) with the CsCl-type structure at 0 GPa.

As shown in Figure 3, there was no band gap in the PDOS of the *Pnma* or *Fd*-3*m-*structured BaAu compounds, which means that the BaAu compounds showed metallic characteristics in the pressure range from 0 to 100 GPa. We further calculated the PDOS of BaAu and CsAu with the CsCl-type structure at ambient pressure and plotted them in Figure 5. Compared with the PDOS of CsAu, the Fermi level of BaAu had a significant upward shift, which caused the Fermi level to pass through the conduction bands and made BaAu show metal characteristics. The movement of the Fermi level was caused by the extra electron introduced from Ba when replacing the Cs element with Ba, which is equivalent to doping electrons. We also performed Bader charge analysis on the CsAu and BaAu compounds. We found that the Au atoms in CsCl-type CsAu and BaAu gained 0.76 and 1.20 *e* at zero pressure, respectively. Considering that Bader charge analysis often underestimates the charge transfers for ionic compounds, we believe that the electron on the 6*s* orbital of Cs atoms was fully transferred and Au atoms had a full-shell structure in the CsAu compound. In contrast, the Au atoms gained more than one electron from Ba atoms and acted as *p*-block elements in the BaAu compound, which can be seen in Figure 5b. These results offer an understandable explanation for the different electronic properties of CsAu and BaAu compounds.

## 4. Conclusions

In summary, we investigated the stabilities, crystal structures, and electron properties of BaAu compounds under pressure by using first-principles calculations. BaAu exhibited metal characteristics under the pressure range of 0–100 GPa. Detailed analysis of the electronic properties showed that the electronic configurations of elements played key roles in determining the electronic properties of gold-bearing compounds. These findings offer a reasonable explanation for the metallic characteristics of BaAu compounds under high pressure and may represent a step forward in understanding the electronic properties of aurides.

## Figures and Tables

**Table 1:** Structural parameters of stable BaAu compounds under high pressure.

Compound	Pressure (GPa)	Space Group	Lattice Parameter (Å)	Atomic Coordinate (Fractional)			
				Atoms	*x*	*y*	*z*
BaAu	10	*Pnma*	*a =* 7.922	Ba	0.317	0.750	0.136
			*b =* 4.705				
			*c* = 6.087	Au	0.040	0.250	0.136
			*α = β = γ* = 90				
BaAu	25	*Fd*-3*m*	*a = b = c* = 7.034 *α = β = γ* = 90	Ba	0.250	0.250	0.250
				Au	0.750	0.750	0.750

## Data Availability

Data sharing is not applicable to this article.

## Supplementary Materials

The following are available online at https://www.mdpi.com/article/10.3390/ma15207381/s1, Table S1: Structural parameters of other BaAu compounds at atmospheric pressure; Figure S1: Calculated electron localization function (ELF) for *Pnma*-BaAu compounds at 0 GPa; Figure S2: Phonon dispersions of BaAu with *Fd*-3*m* symmetry at 100 GPa.
