# Supplementary material for: Crystal Structures and Electronic Properties of BaAu Compound under High Pressure
Source: Materials (Basel). 2022 Oct 21;15(20):7381. doi: 10.3390/ma15207381 (PMC9606986; doi:10.3390/ma15207381)
Supplement: Supplementary file 1 [file materials-15-07381-s001.zip › materials-1923190-supplementary.pdf]

Crystal structures and electronic properties of BaAu compound under high pressure

Bingtan Li[1], Jianyun Wang[1, *], Shuai Sun[2, *] and Hanyu Liu[1]

[1]*State Key Laboratory of Superhard Materials and International Center of Computational Method & Software, College of Physics, Jilin University, Changchun 130012, China*

[2]*Engineering Training Center, Jilin University, Changchun 130012, China*

Table S1 Structural parameters of other BaAu compounds at atmospheric pressure.

Compound	Space group	Lattice Parameter(Å,°)	Atomic coordinate (fractional)			
			Atoms	x	y	z
BaAu	*R*-3*m*	$a = b = 3.609$<br>$c = 17.601$<br>$\alpha = \beta = 90$<br>$\gamma = 120$	Ba	0.000	0.000	0.000
			Au	0.333	0.667	0.167
BaAu	*Fm*-3*m*	$a = b = c = 6.734$	Ba	0.500	-0.500	0.500
		$\alpha = \beta = \gamma = 90$	Au	0.000	0.000	0.000
BaAu	*P*4/mmm	$a = b = 3.698$<br>$c = 4.728$	Ba	0.500	0.500	0.500
		$\alpha = \beta = \gamma = 90$	Au	0.000	0.000	0.000

BaAu	*Cmcm*	a = 4.271<br>b = 12.598	Ba	0.500	0.640	0.250
		c = 5.191<br>$\alpha = \beta = \gamma = 90$	Au	0.500	0.915	0.250
BaAu	$P2_13$	$a = b = c$= 6.457	Ba	0.153	0.347	0.653
		$\alpha = \beta = \gamma = 90$	Au	0.845	0.655	0.345

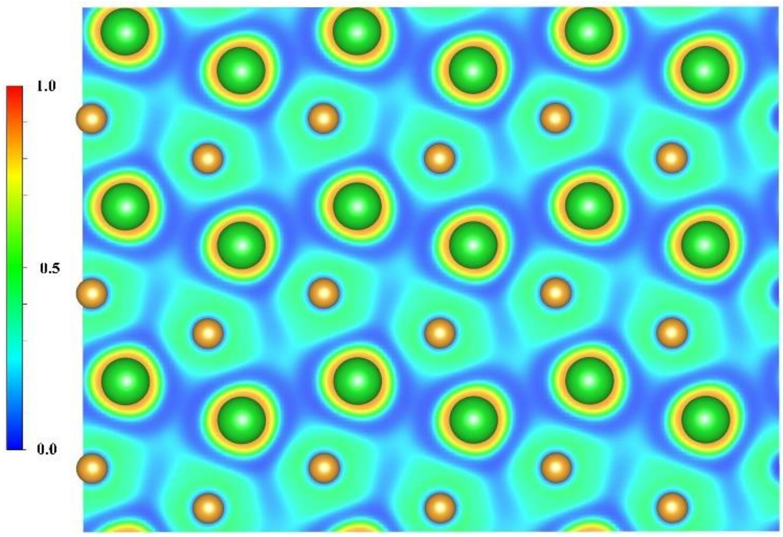

Figure S1. Calculated electron localization function (ELF) for *Pnma*-BaAu compounds at 0 GPa.

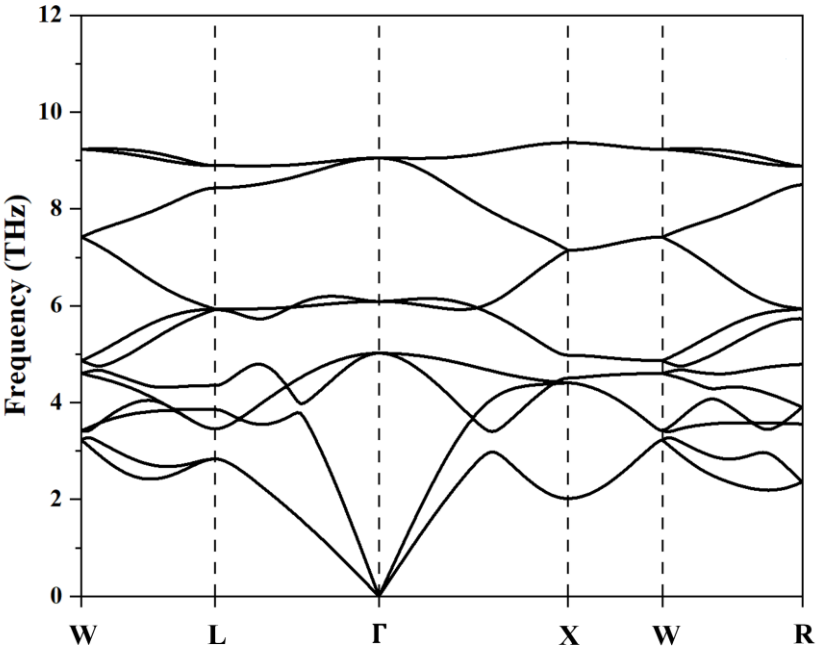

Figure S2. Phonon dispersions of BaAu with *Fd*-3*m* symmetry at 100 GPa.
